# Competency models are no silver bullet for solving health-care managers’ challenges. Perspectives from frontline managers in municipality health care

**DOI:** 10.1108/LHS-09-2025-0156

**Published:** 2026-07-03

**Authors:** Ingrid Marie Leikvoll Oskarsson, Tonje Hungnes

**Affiliations:** Faculty of Business Administration and Social Sciences, Molde University College, Molde, Norway

**Keywords:** Competence mapping, Health-care management, Frontline managers, Municipal health care, Qualitative research, Norway, MCAP

## Abstract

**Purpose:**

This study aims to explore how frontline managers (FLMs) in Norwegian municipal health care perceive the relevance and practical usefulness of the management competency assessment partnership (MCAP) competency model. While competency models are widely promoted to strengthen health-care management and leadership, fewer studies have examined how such models are experienced by FLMs who work under considerable operational and contextual constraints.

**Design/methodology/approach:**

A qualitative study was conducted using three focus group discussions with 14 FLMs from three Norwegian municipalities. Before discussions, participants completed the MCAP questionnaire and reflected individually on its strengths, limitations and relevance. Data were analysed inductively using qualitative content analysis.

**Findings:**

The analysis revealed two interrelated findings. First, FLM perceived MCAP as a relevant and comprehensive model that reflected the formal expectations for their role. Second, they questioned its practical usefulness when considered in relation to everyday work. Large spans of control, broad task scope, time pressure and limited room for manoeuvre constrained managers’ opportunities to develop, prioritise or enact several competencies. This was particularly consequential for interpersonal communication and change management, which were highlighted as the most important competencies by the informants.

**Originality/value:**

This study contributes a conceptual lens for interpreting competency models as espoused theories and contrasting them with the theories-in-use that managers develop to meet everyday operational demands. It shows that competency mapping alone is no silver bullet for strengthening management. Effective competency development requires organisational conditions that enable FLMs to enact and sustain the competencies that models such as MCAP define.

## Introduction

Health-care organisations increasingly use competency models to define the knowledge, skills and abilities required for effective leadership and management (e.g. Gunawan *et al.*, 2020; Kantanen *et al.*, 2017; Liang *et al.*, 2020). Such models are intended to support recruitment, role clarification, self-assessment, professional development and organisational improvement. Evidence links competent health-care managers to lower staff turnover (Nelsey and Brownie, 2012), successful reform implementation (Ayeleke *et al.*, 2018), and improved service quality and organisational performance (Groves, 2011; Kerfoot and Luquire, 2012). At the same time, critics argue that competency models may underrepresent the social, organisational and clinical complexity of health care practice (Bradley *et al.*, 2015).

The Management Competency Assessment Partnership (MCAP) model was developed in Australia to identify health management competencies across different settings. It includes six core competencies supported by 82 associated behavioural indicators and has demonstrated validity across several international contexts (Kakemam *et al.*, 2021; Liang *et al.*, 2018; Howard *et al.*, 2018; Sandhu and Liang, 2021; Ylitalo *et al.*, 2023). MCAP therefore offers a structured way to describe and assess management competencies. However, less is known about how frontline manager (FLM) experience the relevance and usefulness of such a model when it is applied in daily municipal health-care practice. FLMs are described as middle-level managers positioned closest to the production core, who play a pivotal role in enhancing nurse satisfaction, supporting recruitment and retention, and fostering healthy work environments amid an escalating global nursing shortage (Cummings *et al.*, 2018).

FLMs occupy a demanding position between strategic decision-making and daily service delivery. They are expected to translate organisational goals into practice, manage staff, oversee budgets, secure quality and patient safety, and create supportive work environments. These responsibilities are often carried out under resource constraints, heavy workloads and ethical dilemmas that can contribute to moral distress and burnout (Edwin *et al.*, 2023; World Health Organization, 2016; Søreide *et al.*, 2019). Large spans of control can increase role overload and reduce leadership effectiveness (Boned-Galán *et al.*, 2023). Recent research suggests, however, that the relationship between span of control and turnover is complex. In a longitudinal cohort study of FLMs, insufficient control over work and inadequate managerial support predicted turnover, whereas span of control and number of employees did not (Svanström *et al.*, 2025a). This aligns with qualitative evidence showing that FLMs’ ability to manage effectively depends on structural conditions such as resources, information, opportunities for development and support from superiors (Lundin *et al.*, 2025).

In Norway, competency mapping is identified as a strategy for supporting and retaining health-care managers and improving the efficiency of municipal health and care services (Regjeringen, 2021). This policy emphasis reflects persistent challenges in recruiting and retaining qualified managers. Turnover among FLMs is a concern because it threatens organisational stability, reduces continuity, increases costs and may undermine public trust in health services (Cregård *et al.*, 2017; Keith *et al.*, 2021). Three out of four nursing home managers in Norway have considered leaving their positions (Aagestad, 2022), illustrating the urgency of understanding the conditions under which managers work.

Against this background, competency mapping may be useful, but only if the model is meaningful in relation to the work managers actually perform. This study therefore explores how Norwegian FLMs perceive the MCAP competency model in relation to their managerial role and organisational context. The research question guiding this inquiry was: how do FLMs in Norwegian municipal health care perceive the relevance and practical usefulness of the MCAP competency model for their managerial practice and development?

The study has two objectives. Firstly, it investigates how FLMs assess the MCAP as a well-established competency model for health-care management. Secondly, it explores how FLMs reflect on the potential of such a model to strengthen management practice and support professional development in municipal health care.

## Material and methods

### Study setting and design

This study was initiated in response to concerns raised by health-care directors in three Norwegian municipalities regarding difficulties in recruiting and retaining FLMs in municipal health-care services. The directors were interested in measures that could make the FLM role more attractive and support the retention of current managers. One proposed strategy was to examine whether competency mapping could identify management development needs and strengthening managerial support.

A qualitative design was chosen to explore how FLMs perceived the relevance and practical usefulness of MCAP in relation to everyday managerial work. Focus group discussions were considered appropriate because the study aimed to examine how participants collectively reflected on, compared and discussed their experiences of using a competency model within their organisational context (Krueger, 2014). This aligns with Krueger and Casey’s (2002) description of focus groups as suitable for understanding how people think and feel about an issue, idea, product and service, particularly when interaction between participants may generate richer insights beyond individual responses.

The MCAP model had recently been translated and pilot tested in the Norwegian health-care context (Leikvoll Oskarsson *et al.*, 2026). The present study did not aim to psychometrically validate the translated model. Instead, it examined how FLMs experienced its content, relevance and practical usefulness as a competency mapping tool.

### Sampling and recruitment

Participants were recruited from three Norwegian municipalities. The inclusion criteria were employment in a frontline management position in municipal health care and personnel and/or operational management responsibilities. The target group was therefore defined by direct experience with the phenomenon under study: frontline management in municipal health care and the practical relevance of a management competency model.

Health-care directors in each municipality acted as gatekeepers and identified eligible FLMs from their services. They invited managers who met the inclusion criteria and arranged meeting rooms at the participants’ workplaces. Recruitment can therefore be described as purposive and gatekeeper-mediated (Andoh-Arthur, 2019; Palinkas *et al.*, 2015). The research team did not select participants directly and did not have access to information about FLMs who declined or were not invited. This procedure may have introduced selection bias, as directors’ discretion influenced which FLMs were approached. However, the final sample included managers from several municipal health-care service areas, providing variation in workplace context, age and management experience.

A total of 14 FLMs participated in the three focus groups. The groups consisted of three, five and six participants. Krueger and Casey (2002) recommend approximately six to eight participants per focus groups, with enough participants to generate diverse ideas but not so many that participants have limited opportunity to speak. Two groups were close to this recommendation, while one group was smaller. The smaller group was retained because all participants met the inclusion criteria and provided detailed reflections relevant to the research question.

Participants included 12 women and 2 men, aged 28–60 years, with management experience ranging from 0,4 to 18 years. They were employed across seven municipal health-care services areas: nursing home care, home care, services for people with disabilities, housing and habilitation, relief services, intoxication services and daycare. Homogeneity in focus groups was sought primarily through participants’ shared hierarchical position as FLMs, their health and social care background, their municipal health-care setting and their responsibility for personnel and/or operational management. Due to variations in how Norwegian municipalities organise services, each group was established within one municipality. At the same time, the groups were not homogenous in terms of specific service context. FLMs in housing and habilitation, nursing home care and intoxication services may work under different operational conditions. This variation was considered relevant to the research aim because the study explored how MCAP was perceived across municipal health-care contexts, but it is also recognised as a factor that may have shaped the discussions.

An overview of participant characteristics is presented in Table 1.

**Table 1.** Presentation of the informants: *n* = 14, gender, age and years of experience as manager in the health-care sector and current working place

Gender	Age, years	Experience, years	Working place
F	42	6	Nursing home
F	35	6	Home care
M	45	3	Actions for disabled
F	53	12	Home care
F	42	11	Housing and habilitation
F	28	2	Relief service
F	60	13	Housing and habilitation
F	42	2	Nursing home
M	53	18	Intoxication service
F	52	15	Housing and habilitation
F	42	3	Housing and habilitation
F	38	2	Housing and habilitation
F	47	18	Housing and habilitation
F	39	0,4	Daycare

**Source(s):** Authors’ own work

### Data collection

Data were collected in November and December 2024 through three face-to-face focus groups sessions held at the participants’ workplaces. Holding the discussions in familiar workplace settings was intended to support accessibility and reduce practical barriers to participation (Krueger, 2014). Participants were seated in a circle to facilitate eye contact and interaction. Each session lasted approximately 2 h. The first part lasted 45–60 min and consisted of individual completion of the MCAP questionnaire. During this part, participants wrote notes regarding the models’ strengths, weaknesses, relevance and possible shortcomings. This was a preparatory exercise rather than the focus group discussion itself. Its purpose was to ensure that all participants had actively engaged with the MCAP model before the group discussion began.

The second part lasted approximately 60–75 min and constituted the focus group discussion. The discussion followed a semi-structured questioning route developed to address the study aim. Questions were predetermined, open-ended, conversional and sequenced from general to more specific topics (Krueger and Casey, 2002; Krueger, 2014). The guide moved from general reflections on the MCAP to questions about relevance, clarity, usefulness, missing elements and practical application of the competencies.

Participants discussed how they perceived MCAP in relation to their managerial role, whether any competencies or behavioural indicators were difficult to understand or apply, and whether important aspects of their work context were missing. To elicit a broad range of perspectives, the moderator asked for positive, negative and neutral reflections. Follow-up probes encouraged elaboration and clarification, and quieter participants were invited into the discussion. The moderator encouraged participants to respond to each other rather than only to the moderator (Nyumba *et al.*, 2018).

Two researchers were present at each focus group: one moderator and one observer/assistant moderator. The moderator was experienced in qualitative research, focus group discussion methodology and health-care research. The moderator facilitated an open and respectful discussion, kept the conversation focused on the research question, encouraged interaction and ensured that all participants could contribute. The observer/assistant moderator provided information about the study, managed the audio recording, observed group dynamics, documented non-verbal communication and took field notes (Krueger and Casey, 2002).

At the beginning of each session, participants were informed that there were no right or wrong answers, that differing views were welcome, and that the purpose was to learn from their experiences. Furthermore, the recording procedure, confidentiality, voluntary participation and participants’ right to withdraw were explained. All focus group discussions were audio-recorded with participants’ consent.

### Transcription and data material

The audio recordings from the discussion part of the focus group were transcribed *verbatim*. The transcripts included spoken content from participants and the moderator. Pauses, laughter and other verbal expressions were noted when relevant to meaning. Transcription was performed using artificial intelligence-assisted transcription software followed by manual revision by the first author. All transcripts were checked against the audio recordings before analysis to ensure accuracy.

The final data material consisted of 73 pages of transcripts from the focus group discussions, participants’ written notes from the individual MCAP exercise and the observer’s field notes. The written notes contextualised and supplemented the group discussions, while the transcripts were the main analytic material. All transcripts were anonymised before analysis.

### Data analysis

Data were analysed using inductive qualitative content analysis as described by Elo and Kyngäs (2008). This approach was chosen because the study sought to explore FLMs’ perceptions of MCAP in a Norwegian municipal health-care context, an area with limited prior qualitative research.

The analysis was systematic and documented throughout. Firstly, three researchers that had been involved in the data collection read the transcripts and field notes several times to become familiar with the material. Open coding was then conducted by identifying meaning units relevant to the research question and assigning preliminary codes (Elo and Kyngäs, 2008). Coding focused on participants’ descriptions of MCAP, their assessments of its relevance and usefulness, and their reflections on organisational conditions shaping their ability to develop or enact management competencies.

Secondly, the research team compared and discussed the preliminary codes. Coding sheets were developed to organise codes across the three focus groups. Similar and overlapping codes were grouped together, while differences between municipalities and service contexts were discussed to avoid prematurely merging distinct experiences. The two authors of this study then sorted the codes into subcategories and higher-order categories based on similarities and conceptual relationships.

Thirdly, the emerging categories were reviewed against the transcripts, written notes and field notes to ensure that they were grounded in the data. Through this iterative process, 56 initial codes were reduced to 9 main categories with 39 subcategories. Further abstraction resulted in a final structure consisting of 2 main categories, 5 generic categories and 16 subcategories. In the final stage, descriptive summaries were written for each category and subcategory. These summaries were discussed and refined by the authors until agreement was reached on the final categorisation and the illustrative quotations.

### Ethical considerations

The study was approved by the Norwegian Agency for Shared Services in Education and Research (reference number 153223). Permission to conduct the research was also granted by the health-care directors in the three participating municipalities.

All participants received written and oral information about the study before participation. They were informed about the study aim, what participation involved, the voluntary nature of participation, confidentiality, data handling and their right to withdraw at any time without giving a reason. Written informed consent was obtained from all participants. Audio recordings, transcripts and notes were stored in a secure data management system approved for research data.

## Results

**Figure 1.**
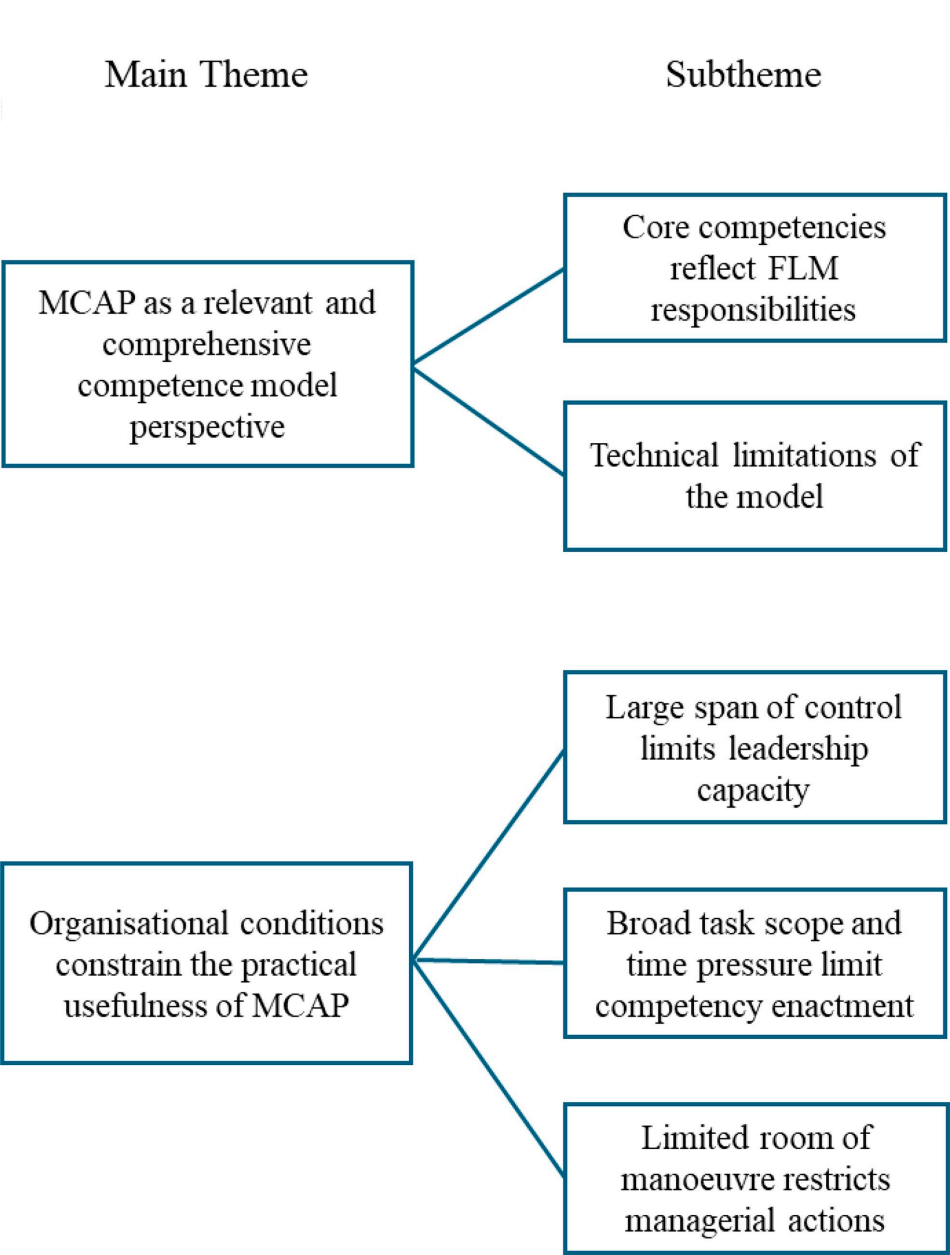
A theme map links 2 main themes about M C A P to 5 subthemes about competencies, limitations and organisational constraint **Source:** Authors’ own work

The analysis revealed a twofold perception of the MCAP competency model among the FLMs. Firstly, when assessed in relation to formal role expectations, MCAP was perceived as a relevant and comprehensive model that captured important managerial competencies. Secondly, when assessed in relation to everyday managerial practice, participants questioned the model’s usefulness because organisational conditions constrained their opportunities to develop, prioritise and enact several of the competencies. These two main themes and their subthemes are presented in Figure 1.

### Management competency assessment partnership as a relevant and comprehensive competency model

The first main theme reflects the FLMs’ assessment of MCAP from an idealised perspective, that is, in relation to the competencies their managerial role is expected to encompass. From this perspective, participants consistently described MCAP as comprehensive, relevant and broadly aligned with municipal health-care management.

### The core competencies reflect frontline manager responsibilities

Participants highlighted the background section and the six core competencies as relevant and clearly described. None of the behavioural items were perceived as irrelevant to the FLM role.

Although not all competencies were discussed in equal depth, several were emphasised. Participants valued the emphasis on operations, administration and resource management, including financial management. They described financial literacy as important for sustainable operations, even though financial decision-making beyond overseeing an allocated budget was not part of their formal role. Risk management, data-informed quality improvement and performance monitoring were highlighted as relevant to daily practice. Interpersonal communication qualities and relationship management were frequently described as among the most critical competencies. Emotional and relational skills were seen as fundamental to workplace dynamics and operational effectiveness. One informant stated: “The most important thing of all is how you tune in and how your relationships with others are. Relational skills pretty much determine how the operation runs”. Participants also valued that the competency descriptions reflected the challenges managers face in balancing workplace demands while maintaining positive relationships with employees.

Competencies related to leading people and the organisation, as well as enabling and managing change, also resonated strongly. Items such as “Empower others to achieve goals” and “Lead, develop and evaluate performance to build an effective team” were described as closely aligned with central FLM responsibilities. Overall, the participants perceived MCAP as clear and well-structured, and its competency descriptions as aligned with the formal demands of frontline health-care management.

### Technical limitations of the model

Despite overall agreement on the relevance of MCAP, participants identified some limitations. The most frequently discussed concern related to the response scales. MCAP includes a five-point importance scale and a seven-point self-assessment scale. The importance scale was perceived as lacking nuance. This was connected to the scale having a neutral midpoint stating the competency as neither unimportant or important, leading the respondents to move from unimportant to somewhat important. Participants suggested that a more neutral or broader scale could better differentiate between competencies. The self-assessment scale also caused uncertainty, particularly at higher levels, where the wording was perceived as implying extensive executive-level experience. Participants suggested more inclusive wording that could capture knowledge-based competence and experience gained in frontline roles. Several participants were also uncertain about the meaning of “guidance’’ in the assessment scale and asked whether informal discussions with colleagues could be considered equivalent.

Another concern was that the questionnaire allowed respondents to assess the importance of each competency but not whether they actually had the opportunity to practice or enact it. Participants further suggested that MCAP lacked flexibility to capture dynamic and context-specific aspects of FLM work. In particular, they pointed to the absence of measures related to span of control, which they viewed as essential for understanding managerial practice. As one participant noted: “A manager with 20 employees […] the quality and the results are much better than one with 70; then there is no time for anything”. Thus, while MCAP was regarded as relevant and comprehensive, participants also identified technical and contextual limitations that affected its practical applicability.

### Organisational conditions constrain the practical usefulness of management competency assessment partnership

The second main theme reflects FLMs’ assessment of MCAP from the perspective of everyday managerial practice. Although participants acknowledged that the MCAP competencies were important, they emphasised that organisational conditions shaped whether they could develop, prioritise and enact these competencies. Participants often introduced their reflections with “It depends on […].” underscoring the contextual nature of the FLM role. Three interrelated organisational conditions were particularly important: large span of control, broad task scope and time pressure and limited room for manoeuvre.

### Large span of control limits leadership capacity

A recurring theme was that large span of control limited participants’ capacity to engage in management activities beyond daily operations. Participants described responsibility for large teams, often including 60–90 permanent employees in addition to temporary and part-time staff. This left limited time for leadership, development work and systematic competency development. One participant questioned the value of competency development when organisational conditions did not allow the competency to be used: “Is it useful to take a course in change management if the organisational conditions do not allow for development work?”

Several participants described large team sizes as normalised within municipal health care: “No one reacts […] managing 70–80 employees is just accepted as the norm”. Others described administrative overload during staff shortages and sickness absence:

The nursing home has 86 permanent employees, but on your list, you have 110-120 [including temporary and part-time staff] […] this number reflects how many phone calls we get and how much we do in our free time.

Participants also discussed the difficulty of exercising relational and developmental leadership when employees worked rotating shifts and were not necessarily present during an ordinary work week.

The emotional burden of managing complex staff dynamics was also highlighted. One participant explained:

Those who quarrel a lot take up so much of your time. And then you don’t have time for the quiet ones – those who might end up on sick leave because they’re worn out.

Participants emphasised that a stable and well-trained workforce could reduce stress, absenteeism and dependence on temporary staff. Although support systems existed, they were not always sufficient: “We have people who can help, but still, following up with staff, dealing with high sick leave […] Some units have absenteeism rates as high as 25%. It’s chaotic”.

### Broad task scope and time pressure limit competency enactment

Participants generally agreed that the competencies included in MCAP reflected the breadth of the FLM role. However, the broad scope of their responsibilities made it difficult to enact these competencies in action. Participants described juggling multiple roles and tasks, often beyond what they considered core managerial responsibilities: “We are janitors, safety nurses, managers […] involved in everything from diaper changes and wound care to handling challenging behaviour and implementing action plans”. Another participant stated:

We must act as psychologists, lawyers concerning working environment laws and collective agreements, and health professionals […] while also being effective project managers, navigating changes and managing it all – it’s overwhelming […] we need to know everything.

Participants expressed frustration that they often had the necessary knowledge but lacked the time and opportunity to apply it: “It’s not necessarily that we lack knowledge – we don’t have the opportunity to apply what we know because the time isn’t there”. This was especially clear in relation to change management: “It’s great that ‘Leading Change’ is being highlighted. We wish we had more time for it, but daily operations consume us”.

Participants described how time pressure and broad task scope challenged the implementation of competency mapping results. Identifying development needs was not enough if work conditions did not allow managers to act on them. One participant argued that improving conditions for FLMs required rethinking how services were organised:

We are not able to support and see everyone […] we may have to look at how management is organised, because I think that many of the challenges lie there […] creating good routines and procedures […] Then employees could have been more secure […] They would know what to do when they were supposed to, and they knew that they would get support when they faced a challenge.

Despite recognising the need for competency development, participants described resource constraints as limiting their ability to make meaningful change:

My manager sees that I work myself to death sometimes, but that doesn’t help when we lack the authority to make meaningful changes […] the real challenge lies in the scarcity of resources and the extent to what our management position should entail […] we are performing the work of six or seven people.

Several participants suggested that some tasks currently handled by managers could be delegated to others, thereby reducing workload and better using staff competencies. One participant concluded: “I’d like to be a nurse manager […] not doing secretarial work”.

### Limited room for manoeuvre restricts managerial action

The third organisational condition concerned participants’ limited room for manoeuvre. FLMs described restricted decision-making authority, particularly regarding budgeting, resource allocation and staffing. These areas were strongly influenced by political decisions, regulations and fixed financial frameworks. One participant explained:

We are responsible for managing decisions that have already been made […] we must adhere to laws and regulations as everyone has the right to health services if they meet the criteria. This makes it challenging to manage within a budget […].

At the same time, some participants described exercising discretion within procedural boundaries: “There is a procedure […] however, some of us prefer to use our own judgment and not follow the procedure to the letter […]”

Staff competency was described as central to whether departments functioned effectively. Participants emphasised that when staff lacked sufficient competence, managerial workload increased: “When everything falls on us, it really depends on the staff you have […] If you don’t have trained professionals – you end up doing all the professional work yourself”. Participants also discussed limited room for manoeuvre in relation to disciplining or replacing employees who did not function well in their roles. Strong employee protections in the Norwegian public sector were described as limiting managers’ ability to address persistent staff-related challenges.

## Discussion

This study explored how FLMs in Norwegian municipal health care perceive the relevance and practical usefulness of the MCAP model for performing their roles. The findings show that FLMs perceived the MCAP in two interrelated and distinct ways. Firstly, the model was seen as relevant and comprehensive, reflecting key FLM responsibilities. Secondly, participants questioned its practical usefulness in everyday managerial work, where organisational conditions constrained their ability to enact and develop these competencies.

### Management competency assessment partnership as a relevant competency model

The first main finding indicates that FLMs perceived MCAP as aligned with formal role expectations. Participants recognised the core competencies and behavioural indicators as relevant, and none of the competencies were described as irrelevant. This supports previous research showing that MCAP captures central health-care management competencies across contexts (Howard *et al.*, 2018; Liang and Kakemam, 2025; Sandhu and Liang, 2021).

Existing research has identified several MCAP competency areas as important for health-care managers, including evidence based practice (Koivunen *et al.*, 2024), organisational knowledge (Vainieri *et al.*, 2019), interpersonal and personal traits and leadership skills (Gunawan *et al.*, 2023) and change management (González García *et al.*, 2020; Gunawan *et al.*, 2020). The present study extends this knowledge by showing that FLMs in Norwegian municipal health care also recognise these competencies as important. Financial management was also considered important, although decision-making beyond managing an allocated budget is not typically central to Norwegian FLM. This may reflect increasing expectations that health-care managers contribute to efficiency and sustainable resource use (González‐García *et al.*, 2021).

At the same time, participants identified limitations. The importance scale made prioritisation difficult because the scale limited the informants’ possibilities to assess the importance levels of the competencies more nuanced. Participants also questioned whether the model captured managers’ opportunities to enact the competencies. This suggests MCAP may be strengthened by including contextual indicators such as span of control, available support, delegated authority and opportunities to apply competencies in practice.

### Organisational conditions and the practical usefulness of management competency assessment partnership

The second main finding shows that organisational conditions shaped participants’ assessment of MCAP’s practical usefulness. Although FLMs regarded the competencies as relevant, they repeatedly emphasised that relevance alone did not ensure enactment. Large spans of control, broad task scope, time pressure and limited managerial autonomy restricted their ability to prioritise leadership, development work, change management and relational follow-up. This finding is consistent with research showing that large spans of control can contribute to role overload, increased workload and reduced leadership effectiveness (Boned-Galán *et al.*, 2023).

The broad task scope described by participants further illustrates this point. FLMs were responsible for clinical, administrative, relational, legal and operational tasks. This breadth made all MCAP competencies appear relevant, but difficult to prioritise or apply. Participants did not primarily describe a lack of competence; rather, they described lack of time, support and organisational capacity. This distinction is important because it suggests that competency mapping alone may identify development needs without addressing barriers to enactment.

Limiting room for manoeuvre was another central constraint. Participants described restricted authority over budgeting, staffing and service development. Although responsible for implementation and service quality, they had limited influence over the conditions needed to deliver them. This aligns with recent research suggesting that managerial control, autonomy and support may be more important predictors of turnover than team size alone (Svanström *et al.*, 2025b; Svanström *et al.*, 2025a). The findings therefore support a nuanced understanding of FLM challenges: span of control matters, but its consequences are closely connected to available authority, support and resources.

### Management competency assessment partnership in the realities of municipal health-care management

The two main findings can be understood through Argyris and Schön’s (1978) concepts of espoused theory and theory-in-use. Espoused theory refers to formal explanations, ideals and principles that individuals or organisations articulate about their actions, whereas theory-in-use reflects the implicit logic that guides action in practice (Argyris, 1999; Argyris and Schön, 1978). From an espoused theory perspective, MCAP represent an idealised description of the competencies health-care managers should possess and enact. Participants’ positive evaluations of MCAP therefore reflect their recognition of the formal FLM expectations.

When participants discussed everyday work, a theory-in-use perspective became visible. Their accounts showed how actual managerial practice was shaped by organisational pressures, limited time, large staff groups, administrative overload and constrained autonomy. This helps explain why a theoretically robust model such as MCAP may be embraced conceptually while still being difficult to apply in practice. The issue is not that the competencies are irrelevant, but that the organisational conditions often prevent FLMs from using them. This tension between formal expectations and everyday practice is central to the contribution of the study. It suggests that competency models risk functioning as descriptions of an ideal managerial role unless they are accompanied by attention to the organisational conditions that enable or constrain managerial action.

### Implications for competency mapping and managerial development

The findings suggest that competency mapping alone is insufficient to address challenges related to health-care management, service efficiency or managerial turnover. If competency models are applied in isolation, organisations may focus primarily on identifying and correcting individual competency gaps. This risks producing single-loop learning, where the response is to develop individual managers without questioning the organisational conditions that shape managerial behaviour.

A more useful approach would involve double-loop learning, where organisations use competency mapping not only to ask what competencies FLMs need, but also what structural conditions must be changed for those competencies to be enacted in practice (Argyris and Schön, 1978). In this study, such conditions included span of control, task scope, workload, delegation, decision-making authority, administrative support, staffing responsibilities, sickness absence follow-up and support from senior management. These contextual factors shaped how FLMs understood their leadership role, which competencies they could prioritise in everyday work, and which competencies they perceived as most important for managing health-care services.

This distinction is important because formal competency models represent an espoused theory of health-care management competence: they describe what managers are expected to know and do. However, FLMs’ managerial practice is also shaped by theories-in-use, meaning the practical assumptions, routines and constraints that guide action in everyday work. For example, a competency model may emphasise leadership, staff development and improvement work, while FLMs’ theories-in-use may be shaped by the need to handle staffing shortages, sickness absence, administrative demands and immediate operational problems. In such situations, competencies that are formally emphasised may not be enacted, not because FLMs lack them, but because organisational conditions limit their use.

**Figure 2.**
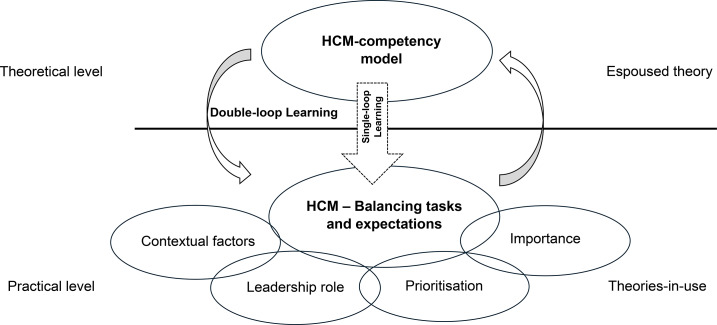
A learning framework diagram links an H C M competency model with practical H C M tasks, contextual factors and learning loops **Source:** Authors’ own work

To support double-loop learning, organisations therefore need to examine the relationship between espoused competency models and the theories-in-use that shape FLM practice. This means using competency mapping not only to identify individual development needs, but also to question whether current organisational structures enable FLMs to enact the competencies expected of them. In this way, the espoused theory represented by MCAP can be adjusted considering the realities of frontline managerial practice, as illustrated conceptually in Figure 2.

These findings have practical relevance because they connect competency development to the structural realities of health-care management (HCM). The results suggest that the question is not only whether FLMs possess the right competencies, but whether their organisations create conditions in which those competencies can be enacted. A competency model can support role clarification, self-assessment and professional development, but it may have limited impact if managers lack time for staff follow-up, authority to adjust resources or support from senior management. Using MCAP as a basis for dialogue between FLMs and senior managers may therefore help organisations identify both individual development needs and organisational barriers to effective management. This dual use is important because it shifts competency mapping from an individual assessment exercise to a tool for organisational learning.

For practice, competency mapping should be combined with organisational measures that address workload, administrative support, span of control, delegation, decision-making authority and access to managerial support. Support functions related to sickness absence follow-up, staffing, administrative tasks and service coordination may be necessary if FLMs are to prioritise leadership, staff development and improvement work. Competency development may therefore be most effective when implemented as part of a broader organisational strategy rather than as an individual-level intervention.

### Quality and credibility

Several steps were taken to strengthen the quality and credibility of the study. Firstly, the design followed established focus group recommendations, including the recruitment of participants with direct experience of the phenomenon, a semi-structured questioning route, audio recording, field notes and systematic analysis. All participants held FLM positions in municipal health care and could therefore provide experience-based reflections on the relevance and practical usefulness of MCAP.

Secondly, credibility was supported through the combination of individual preparation and group discussion. Completing the MCAP questionnaire before discussions ensured that all participants had engaged with the model. The subsequent focus group discussions allowed participants to compare, challenge and elaborate on each other’s reflections, generating richer data than individual responses alone.

Thirdly, the analysis was conducted systematically using inductive qualitative content analysis. The researchers moved from open coding to grouping, categorisation and abstraction and final categories were discussed and refined within the research team. This supported dependability by making the analytic steps traceable. Illustrative quotations strengthen confirmability by showing how interpretations were grounded in participants’ accounts. Field notes were used to contextualise the transcripts and support interpretation of group dynamics and non-verbal interaction. The involvement of a third researcher in the focus groups and initial analysis also contributed to reflexive discussion of emerging categories and reduced the risk that findings reflected a single interpretation.

Some limitations should nevertheless be acknowledged. Recruitment was purposive and gatekeeper-mediated, as health-care directors identified and invited participants. This may have influenced who participated and may have excluded managers with different or more critical experiences. One focus group was smaller than commonly recommended which may have influenced group dynamics. The groups were homogeneous in terms of hierarchical role, sector and broad professional background, but not in specific service context. This variation limits claims of uniformity, but it also strengthens transferability by showing that similar concerns about workload, span of control and limited room for manoeuvre were raised across service settings.

## Conclusion

This study confirms MCAP’s value as a relevant and comprehensive competency model for FLMs in Norwegian municipal health care. At the same time, it shows that the practical usefulness of competency models may depend on the organisational conditions in which managers work. The competencies described in MCAP were perceived as important, but participants’ ability to enact them was constrained by large spans of control, broad task scope, time pressure and limited room for manoeuvre.

This study therefore suggests that competency mapping should not be treated as a stand-alone solution to challenges in health care. Rather, it should be integrated with organisational development efforts that provide FLMs with the time, resources, support and authority needed to practice and develop the competencies that competency models define. Competent FLMs are essential for effective and sustainable health-care services, but competence alone cannot compensate for organisational conditions that prevent managers from leading, developing staff and improving services.

The contribution of this study lies in framing competency models as espoused theories and contrasting them with the theories-in-use that managers develop to meet real-world conditions. By foregrounding the experiences of FLMs, the findings provide guidance for policymakers and organisational managers seeking to strengthen health-care management in ways that are both competency based and context sensitive.

## Limitations

This study was conducted among FLMs in three municipalities within a single Norwegian county. Municipalities in Norway have substantial autonomy in how they organise primary health-care services and differ in economic conditions and resource levels. These contextual variations likely influence the structural and operational realities under which FLMs work. Consequently, the findings may not fully represent FLMs across Norway.

The findings should therefore be read as analytically transferable rather than statistical generalisable. Their relevance lies in the detailed account of how a well-established competency model is interpreted by managers who face substantial operational demands. Readers in other health-care systems may judge transferability by comparing their own management structure, staffing models and degrees of managerial autonomy with those described in this study.

Future research could compare FLMs across diverse municipalities or counties to explore how different organisational and economic conditions shape perceptions of health-care management competencies. The proposed interplay, and occasional tension, between espoused theory and theory-in-use in health-care management also warrants further investigation. Such inquiry could help refine theoretical understandings of how frontline management competencies are defined, enacted and supported in practice.
